# Acute Immunoallergic Hepatitis in the Presence of Crohn’s Disease: A Case Presentation and Review of the Literature

**DOI:** 10.7759/cureus.28174

**Published:** 2022-08-19

**Authors:** Brianna R Taylor, Kwame Opoku, Izi Obokhare

**Affiliations:** 1 Surgery, Texas Tech University Health Sciences Center, Amarillo, USA; 2 General Surgery, Texas Tech University Health Sciences Center, Amarillo, USA

**Keywords:** maculopapular rash, fluoroquinolone, colorectal, hepatitis, autoimmune

## Abstract

In this case presentation, we discuss a patient with acute immunoallergic hepatitis after fluoroquinolone use. The patient presented with a complex perianal abscess two months after the initial procedure for a perianal abscess. He was placed on broad-spectrum antibiotics and underwent fecal diversion with partial colectomy to aid in wound care and diagnosis of Crohn’s disease. Acute hepatic insufficiency and ascites in the presence of a maculopapular rash suggested an immunoallergic reaction. The patient’s condition improved after discontinuation of the offending drug and utilizing intravenous steroids. The diagnosis of acute immunoallergic hepatitis was made based on clinical suspicion and a careful review of the patient’s medical records.

## Introduction

Fluoroquinolones are a widely used antimicrobial drug with high bioavailability and low risk for adverse events. These drugs act by directly inhibiting DNA synthesis, thus exhibiting bactericidal properties. While the array of adverse events has been widely reported in the literature, some are less frequent than others. Some common events may include kidney injury, central nervous system disturbances, Q-T interval prolongation, and gastrointestinal tract offense. Rare occurrences include acute tendon rupture, psychosis, cardiotoxicity, and photosensitivity [1]. Transaminitis may occur as well, with fulminant hepatitis occurring exceptionally rarely. In this report, we discuss a case of fulminant hepatitis in a young male patient with Crohn’s disease who received intravenous (IV) ciprofloxacin postoperatively following incision and drainage of a recurrent perianal abscess.

## Case presentation

A 23-year-old male was admitted for complaints of severe anorectal pain. On physical examination, he was found to have a chronic perianal abscess. Computed tomography (CT) of the abdomen/pelvis showed evidence of perirectal fluid within the left perineal area extending both caudally and posteriorly with air tracking into the scrotum and base of the penis, raising suspicion of Fournier’s gangrene. CT also showed focal collection within the left hemipelvis adjacent to the rectum and distal sigmoid colon as well as thickening of the distal sigmoid colon extending into the rectum. The patient underwent incision and drainage of the perirectal abscess by general surgery and underwent cystoscopy and incision into the superficial left hemiscrotum by urology. Postoperatively, intravenous Invanz and Zyvox were started. Blood cultures returned negative, and anaerobic cultures of the abscess returned positive for *Bacteroides ovatus*. The patient was discharged on postoperative day eight.

**Figure 1:**
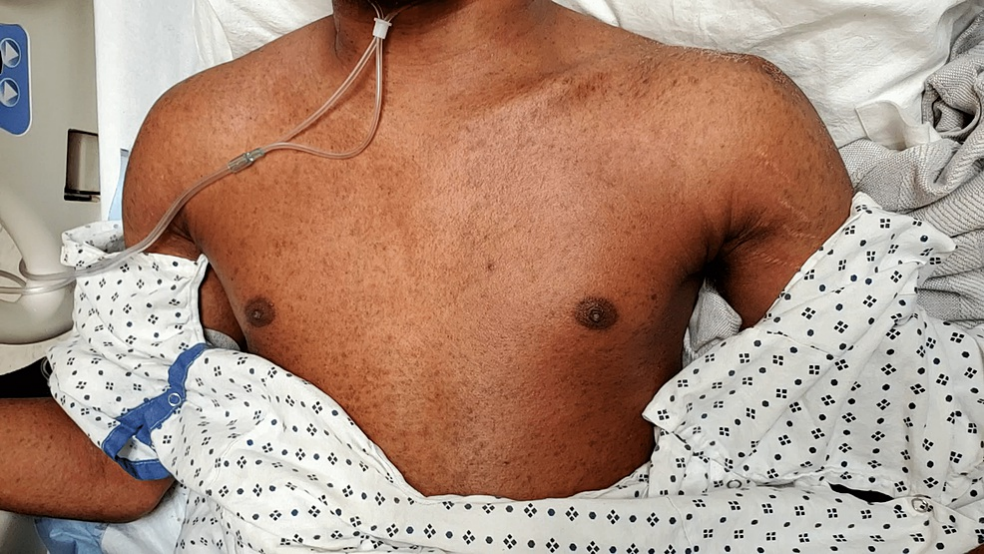
Maculopapular rash present two days after the administration of fluoroquinolone.

**Figure 2:**
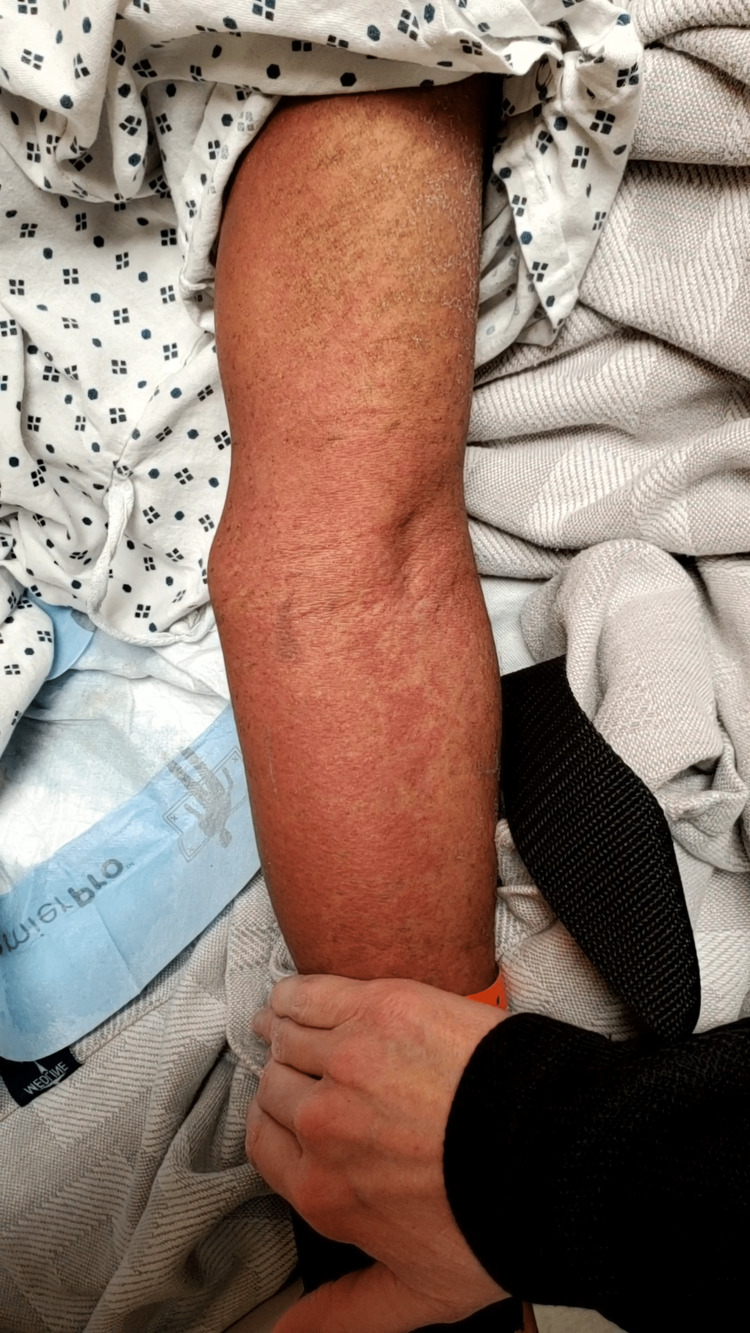
Maculopapular rash present two days after the administration of fluoroquinolone.

Two months later, the patient was readmitted for severe anorectal pain. The patient began having increased perirectal pain and generalized weakness three days prior to presentation. On physical examination, a persistent large open wound was noted in the left aspect of the perirectal area from the prior surgery with purulent drainage. CT of the abdomen/pelvis showed diffuse inflammation and thickening of the rectal/sigmoid colon wall and soft-tissue swelling of the left gluteal area extending to the perirectal area. Laboratory examination showed mildly elevated liver function test (LFTs) (aspartate transaminase (AST) 60 U/L, alanine transaminase (ALT) 71 U/L, alkaline phosphatase (ALP) 240 U/L) with a negative acute hepatitis panel. The patient underwent incision and drainage of the perineal area by general surgery. Postoperatively, the patient was started on IV ciprofloxacin. On postoperative day two, the patient was febrile and tachycardic, and a diffuse, pruritic, raised sandpaper rash was found over the patient’s torso and extremities (Figures 1, 2). Laboratory examination showed significantly elevated LFTs (AST 478 U/L, ALT 346 U/L, ALP 305 U/L, total bilirubin 1.27 mg/dL, direct bilirubin 0.99 mg/dL) and a white blood cell (WBC) count of 20.3. Ciprofloxacin was discontinued, IV Zyvox was initiated, and IV Zosyn remained. Two days later, a repeat CT of the abdomen/pelvis showed calculus cholecystitis and thickened colon. The patient then underwent laparoscopic cholecystectomy, laparoscopic sigmoid colectomy with end colostomy, perianal wound washout, and colonic biopsy to evaluate for inflammatory bowel disease and to provide easy care for the complex perianal wound. On postoperative day three, the patient’s rash worsened and laboratory examination showed continued elevation of LFTs (AST 988 U/L, ALT 740 U/L, ALP 324 U/L, total bilirubin 1.99 mg/dL) and a WBC of 37.1. Intravenous Solu-Medrol and diphenhydramine were then initiated. Two days later, the patient’s rash began to lessen and his reported pruritus began to subside, with LFTs showing AST 435 U/L, ALT 715 U/L, ALP 585 U/L, and total bilirubin 1.08 mg/dL. A colonic biopsy also showed results consistent with Crohn’s disease, and prednisone was initiated. The patient’s rash continued to improve over the hospital course, and the patient was discharged with LFTs showing AST 408 U/L, ALT 847 U/L, ALP 330 U/L, and total bilirubin 0.56 mg/dL.

## Discussion

Fluoroquinolones, such as ciprofloxacin, are known to cause serum liver enzyme elevations in approximately 1-3% of cases [2,3]. Typically, these elevations are transient, asymptomatic, and self-resolving; however, ciprofloxacin has been linked to more severe cases of hepatotoxicity, rare instances of fatality, and vanishing bile duct syndrome [4]. Drug-induced immunoallergic hepatic reactions classically present with signs of acute liver damage as well as signs of hypersensitivity, such as fever, rash, pruritus, and eosinophilia. The onset is generally fast, typically occurring within two days to two weeks, with resolution commonly within two to four weeks upon cessation of the drug. The pattern of enzyme elevation identifies the injury type, ranging from cholestatic injury showing isolated elevated ALP more than two times the upper limit, hepatocellular injury showing a >5:1 ratio of ALT to AST, or mixed, falling anywhere in between [5,6].

When deciding whether a liver injury is drug-induced, it is important to consider some key factors: time of onset after administration of the drug, time to recovery after stopping the drug, injury pattern, recurrence with re-exposure to drug, whether or not the drug in question is known to cause acute liver injury, and clinical presentation to rule out other etiologies with similar laboratory patterns [7]. This may include acute hepatitis infection, stress to a cirrhotic liver, etc. There is no way to directly test if ciprofloxacin causes immunoallergic hepatitis, and it should therefore be a diagnosis of exclusion. Some human leukocyte antigen associations have been made with some forms of immunoallergic hepatitis, but no clear mechanisms are currently understood [3]. Symptomatic treatment upon cessation of the drug is generally adequate; however, steroids may be implemented for more severe cases. Patients who experience any degree of drug-induced hepatitis due to ciprofloxacin should be inquired about previous reactions to fluoroquinolones and advised to avoid the drug class altogether [8,9].

## Conclusions

This was a patient who was exposed to fluoroquinolones while in sepsis from a complicated perianal wound and an acute exacerbation of Crohn’s disease. In most patients, the diagnosis is difficult to achieve due to other confounding factors. However, a rapid diagnosis is made based on a careful evaluation of the patient’s clinical condition, current medications, and the presence of acute liver dysfunction. Rapid cessation of the offending drug and IV steroid therapy is the mainstay of treatment. Healthcare providers taking care of patients with unexplained acute liver insufficiency and a rash should consider this diagnosis.
